# Brain-derived neuerotrophic factor and related mechanisms that mediate and influence progesterone-induced neuroprotection

**DOI:** 10.3389/fendo.2024.1286066

**Published:** 2024-02-26

**Authors:** Meharvan Singh, Vignesh R. Krishnamoorthy, Seongcheol Kim, Saira Khurana, Heather M. LaPorte

**Affiliations:** Department of Cell and Molecular Physiology, Stritch School of Medicine, Loyola University Chicago, Maywood, IL, United States

**Keywords:** progesterone, brain-derived neurotrophic factor (BDNF), microRNA (miRNA), neuroprotection, stroke, brain

## Abstract

Historically, progesterone has been studied significantly within the context of reproductive biology. However, there is now an abundance of evidence for its role in regions of the central nervous system (CNS) associated with such non-reproductive functions that include cognition and affect. Here, we describe mechanisms of progesterone action that support its brain-protective effects, and focus particularly on the role of neurotrophins (such as brain-derived neurotrophic factor, BDNF), the receptors that are critical for their regulation, and the role of certain microRNA in influencing the brain-protective effects of progesterone. In addition, we describe evidence to support the particular importance of glia in mediating the neuroprotective effects of progesterone. Through this review of these mechanisms and our own prior published work, we offer insight into why the effects of a progestin on brain protection may be dependent on the type of progestin (e.g., progesterone versus the synthetic, medroxyprogesterone acetate) used, and age, and as such, we offer insight into the future clinical implication of progesterone treatment for such disorders that include Alzheimer’s disease, stroke, and traumatic brain injury.

## The biology of progesterone

Progesterone is a natural progestin and an important gonadal hormone synthesized in mainly in the ovaries of females and both the adrenal cortex and testes of males. Despite the fact that progesterone levels are overall higher in females, it is known that there is recorded similarities between male progesterone levels and those of females specifically during the follicular phase in female menstrual cycle, indicating a definite and important role of progesterone in males (1). Historically, progesterone has primarily been considered with regards solely to the reproductive functions. For example, it plays vital role in not only female fertility and embryo implantation, but also in the maintenance of the uterus and production of inflammatory mediators in the uterine cavity during pregnancy to prevent miscarriage or preterm labor (2). And though our knowledge of progesterone and related progestins with regards to their role in reproductive biology is quite extensive, we also now appreciate there are rather important effects from progesterone in multiple organ systems including the brain, in which progesterone has demonstrated protective effects. The mechanisms associated with the protective effects of progesterone are complex, and as suggested below, may require the right complement of progesterone receptors (e.g., the classical progesterone receptor (PR) and membrane progesterone receptors), may involve the simultaneous influence on multiple cell types (e.g., glia and neurons), as well as regulation of certain classes of growth factors (e.g., neurotrophins).

## The receptor pharmacology of progesterone

Brain regions such as the cerebral cortex and hippocampus express not just the classical progesterone receptors (PR) (3, 4), but also express membrane progesterone receptors (see (5) for review). The “classical” PR is generally described as a nuclear transcription factor, which when bound to specific progesterone response elements (PRE) within the promoter region of target genes, regulates the transcription of such genes. PR-A and PR-B, the two main isoforms of the classical PR, are transcribed from the same gene, and transcription is usually estrogen-dependent. A third isoform, known as PR-C, also exists, but its exact function is unclear (6).

Recently it has been proposed that there are membrane receptors for progesterone. While it has only been recently that the membrane-associated progesterone receptors have been cloned, they have been suggested for years originating from the knowledge and observation of specific and displaceable binding sites found in preparations of synaptosome membranes (7, 8). Zhu et al., discovered a novel membrane-associated progesterone receptor which is termed mPR (9) that is predicted to couple to Gi/o class of G-proteins (10). Additional progesterone membrane receptors include Progesterone receptor membrane component 1 (Pgmrc1) (11–13) which is implicated in abundant features of cellular function ranging from the regulation of reproductive behavior (11), steroidogenesis (14), growth regulation of triple-negative breast cancer (15), to neuronal development (16) and potentially neuroprotective effects of progesterone as described above.

Pgrmc1’s involvement in the cellular functions associated with cytoprotection may be attributed to its role as a positive regulator of many cytochrome P450 catalyzed reactions that are imperative for intracellular sterol metabolism (17). The Singh laboratory has previously demonstrated that, through our experimental conditions showing progesterone-induced neuroprotection, the classical PR, mPR-alpha, mPR-beta and Pgrmc1 are expressed, while we recently determined that progesterone’s ability to elicit an increase in Brain-derived neurotorphic factor (BDNF) expression is dependent on the classical PR (18). With respect to Pgrmc1, however, selective knockdown completely abolishes the ability of progesterone to elicit an increase in BDNF release (19), an effect that we believe to be critical to progesterone’s neuroprotective effects (20). Based on these observations, and at least from the standpoint of neuroprotection, we suggest that the classical PR and the Pgrmc1 are critical, although perhaps not exclusive, mediators of progesterone’s effects on cell viability.

## Progesterone and neuroprotection

Progesterone has been reported to be protective against a variety of insults relevant to brain aging or indeed, various neurodegenerative diseases, that include stroke, traumatic brain injury (TBI), stroke and Alzheimer’s disease (AD). For example, progesterone, at physiologically relevant concentrations, has been shown to significantly ameliorate oxidative and/or excitotoxic injury resulting from glutamate treatment (20–23), glucose deprivation (24), as well as FeSO_4_- and amyloid β-peptide–induced toxicity (24).

With regards to animal models of stroke, progesterone has been shown to be protective, as exemplified by the study by Jiang et al., which illustrated that treatment with progesterone prior to middle cerebral artery occlusion (MCAO) resulted in a significant reduction in cerebral infarction as well as the functional impairments that resulted from the occlusion (25). Interestingly, administration of progesterone following ischemia was also found to be protective (26–28), and resulted in improvements in various functional outcomes, including the rotarod test, and adhesive-backed somatosensory and neurological scores (29). Progesterone has even been shown to provide protection against ischemia-induced visual impairments, as shown by the work of Allen et al. (2015), who demonstrated that post-ischemic administration of progesterone protected against MCAO-induced retinal ganglion cell (RGC) loss, glutamine synthetase upregulation, and glial fibrillary acidic protein (GFAP) upregulation (30). The protective effects of progesterone following insult suggest that both rapid/immediate and long-term mechanisms of progesterone action may underlie the protective effects of progesterone.

In experimental models of traumatic brain injury (TBI), progesterone is also protective. In such models, progesterone administration has been shown to reduce cerebral edema for up to 24 hours following injury. Further, progesterone was found to reduce complement factor C3, glial fibrillary acidic protein (GFAP), and nuclear factor kappa beta (NFκB) in a rodent model of medial frontal cortex impact injury (31), all of which can be interpreted as protective mechanisms. Progesterone not only facilitates cognitive improvement while reducing secondary neuronal loss caused by edema in ovariectomized female rats after TBI, but also elicits similar effects in male rats (32). Lipid peroxidation was also decreased following treatment with progesterone when administered post-TBI in male rats (33).

The neuroprotective effects of progesterone have also been investigated in the context of experimental systems that simulate the pathology of such neurodegenerative diseases as Alzheimer’s disease (AD) and Parkinson’s disease (PD). For instance, a study by Qin et al. (2015) demonstrated the neuroprotective properties of progesterone against amyloid beta (Aβ)_25-35_-mediated neuronal cell death by alleviating mitochondrial membrane potential loss (34). The neuroprotective effects of progesterone were also noted in animal models of AD, where progesterone improved cognitive performance and glucose uptake in neurons in two separate animal models of AD (35, 36). The protection afforded by progesterone seemed to also be generalizable to other models of neurodegeneration, including Parkinson’s disease. For example, progesterone elicited neuroprotective effects in the murine 1-methyl-4-phenyl-1,2,3,6-tetrahydropyridine (MPTP) model of Parkinson’s disease. Of note, and similar to that described in an animal model of stroke, progesterone was protective at administration time points both pre- and post-MPTP treatment (37, 38).

While the anatomical focus of the brain disorders/diseases referenced above were the hippocampus and cortex, it is worth noting that progesterone has also been shown to be protective in other regions of the central nervous system. For instance, work by Thomas et al., highlighted the beneficial effects of progesterone on spinal cord contusion injuries, by showing a reduction in the size of the lesion and a prevention of secondary neuronal loss with progesterone treatment (39). Additionally, progesterone’s protective actions have been shown in the Wobbler mouse, an animal model of spinal cord degeneration, where progesterone treatment promoted morphological and functional recovery (40, 41). Re-myelination can also be induced by progesterone as evidenced by the increased expression of myelin proteins in the damaged sciatic nerves of both young adult rats and old (22-24 months of age) males (42). Based on these findings, progesterone may be of potential therapeutic benefit in diseases where demyelination is an important component that contributes to pathogenesis.

While the studies described above were derived from animal models and cell or tissue culture models, it is noteworthy that a phase II, randomized, double-blind, placebo-controlled clinical trial assessing the efficacy of progesterone treatment for acute traumatic brain injury yielded promising results. Data from this study suggested that progesterone treatment can improve functional recovery, at least in those with moderate, but not severe, traumatic brain injury (43–47). However, the results from the Phase III study (ProTECTIII) failed to corroborate earlier findings, potentially due to suboptimal dosing (48). Assessment of “early dosing” with progesterone, as conducted by Wright et al. (2014), also failed to show a benefit of progesterone administration over control despite treatment within 4 hours of injury (49). Given that TBI in the clinical setting is heterogeneous, unlike the very reproducible injury that is sustained in pre-clinical (animal) models of TBI (which have consistently shown a protective effect of progesterone), the protective effects of progesterone may be evident only in a subset of TBI patients. Indeed, studies like that by Soltani et al. (2017), which focused on diffuse axonal injury (50), extend the earlier suggestion that progesterone may be protective in patients with TBI.

Many studies that have investigated the protective effects of progesterone have done so within the context of estrogen treatment. More specifically, a significant proportion of these studies have evaluated the protective effects of estrogen alone in comparison to combined treatment with estrogen and progesterone. More recently, however, researchers have addressed the influence of progesterone alone. Although growing evidence suggests that treatment with progesterone alone is neuroprotective, the influence of progesterone on estrogen’s neuroprotective effects is more equivocal. Some studies suggest that progesterone does not interfere with the effects of estrogens (E) (21, 51, 52), while other studies have argued that progesterone or synthetic progestins antagonize the effects of estrogen (53–58). Specifically, the work of Murphy and Segal demonstrated that progesterone antagonizes the effect of 17β-estradiol (E2) on hippocampal spine density (59). Additionally, McEwen and Woolley showed that in both adult and developing brains, progesterone contributed to the loss of hippocampal spines and spine synapses noted across the estrous cycle (60), although progesterone did result initially (within the first 6 hours) in an increase in hippocampal dendritic spine density (61). In contrast, a positive effect of progesterone, similar to that of E2 in the hippocampus of a rat stroke model, was reported by Zhao et al. (62), while Foy et al. (63) described that progesterone enhanced long-term potentiation (LTP) and long-term depression (LTD) in rat hippocampus. Future studies will help clarify the biological basis of this apparent discrepancy, which may be the result of multiple factors, that include the experimental model used (reflecting the types of receptors expressed in the model), the chosen concentrations/doses of progesterone, the timing of the progesterone relative to that of estrogen, the timing of progesterone relative to the insult, or potentially, regional differences in the effects of combined estrogen and progesterone.

## BDNF as a neuroprotectant

BDNF belongs to the family of neurotrophins (that include NGF, NT-3 and NT4), and plays a vital role in maintaining brain health by supporting cell viability and synaptic plasticity (64, 65). The initial synthesis of BDNF occurs in both neurons and glia (66, 67) as a glycosylated precursor (pre-pro-BDNF), which is processed into a 35 kDa pro-BDNF, and can then be converted into the 14 kDa mature BDNF intracellularly or extracellularly (68, 69). Released BDNF can exert its functions on target cells by binding to TrkB (a tropomysin related kinase family (Trk) of receptors) or p75 neurotrophin receptor (p75^NTR^) receptor (67, 68, 70, 71). Differentiating the effects of pro- versus mature BDNF is of high importance as they often exert contrary biological functions: while mature BDNF binds to the TrkB receptor to influence neuronal survival, differentiation, and promote long-term potentiation (LTP), pro-BDNF binds preferentially to p75^NTR^ and can promote neuronal apoptosis and long-term depression (LTD) (see (69) and references cited therein), particularly in the absence of TrkB. Furthermore, it is hypothesized that neuronal dysfunction or atrophy that occurs as a consequence of aging or age-associated diseases may result not only from a decrease in mature neurotrophin expression or function (72–75), but potentially also from *increased* accumulation of the pro-neurotrophins. Consistent with this premise, Fahnestock and colleagues have described that the pro-NGF, the pro-neurotrophin for NGF, is increased in brains from individuals diagnosed with AD (76).

As suggested above, there is strong evidence to support the role of BDNF in synaptic plasticity and cognitive function (77, 78), and as such, alteration in its function and/or expression has been implicated in the pathophysiology of aged-related neurodegenerative diseases including AD and PD (79–81), and conversely, restoring BDNF expression and/or function may be therapeutic. In stroke, BDNF has been largely shown to play a protective role. The delivery of BDNF has been shown to promote tissue reparative processes in various animal models of stroke (82, 83) and interventions that improve functional recovery are often associated with increased BDNF levels in the peri-infarct area. For example, Lazarovici et al., reported that pituitary adenylate cyclase activating peptide (PACAP) is protective in a rat model of stroke by inducing BDNF expression and release, as well as activating TrkB receptor (84). Ishrat et al., also showed that the protective effects of progesterone are mediated by BDNF, helping to reduce ischemic lesion size and edema in rats experiencing permanent focal cerebral ischemia (85). Furthermore, gene delivery of BDNF, using a recombinant adeno-associated virus (rAAV), decreased cell death in a rat focal ischemic lesion (86). Conversely, attenuating BDNF levels or its effects following cerebral ischemia often reduces recovery of function (87, 88). It is noteworthy that BDNF is also produced in non-neuronal cells (such as astrocytes, microglia and endothelial cells) after ischemic stroke (89), and therefore these cells may contribute significantly to the BDNF-dependent recovery. BDNF has also been implicated as a neuroprotectant in TBI (for review, see (90) and references cited therein). In the hippocampus, conditional knock out of BDNF increased death of adult-born immature neurons following TBI (91). Conversely, therapeutic improvement and recovery of function after TBI is associated with an induction of BDNF and its associated proteins (92, 93). Changes in BDNF signaling have also been implicated in Alzheimer’s disease and work by Zheng et al., 2010, shows that Aβ reduces mature BDNF expression *in vitro* (94). Conversely, BDNF gene delivery has shown to reverse synapse loss and protect against neuronal death of entorhinal neurons in mouse models of AD (95).

The mechanisms by which BDNF-mediated protection occurs may be elicited through multiple mechanisms including the activation of specific signaling pathways, including the MAPK and phosphoinositide inositol-3 kinase (PI-3K) pathways. For example, exogenous BDNF protects primary cortical neurons from apoptosis in a dose-dependent manner (96). While BDNF increases the phosphorylation of PI3K and Ak Strain Transforming (Akt) (as an indicator of their activation), pharmacological inhibition of the PI-3K pathway by LY294002 prevents the neuroprotective effects of BDNF in primary cortical neurons (96). Likewise, extracellular-signal regulated kinase (ERK)1/2 phosphorylation/activation is significantly increased by BDNF, while pharmacological inhibition of the ERK1/2 pathway by PD98059 greatly reduces BDNF’s neuroprotective effects. In addition, direct administration of BDNF, via intracerebroventricular administration, in postnatal day 7, rats resulted in phosphorylation of ERK1/2 and Akt within minutes (97), while pharmacological inhibition of ERK inhibited the ability of BDNF to block hypoxia/ischemia-induced caspase-3 activation and tissue loss (97). Based on progesterone’s ability to rapidly activate both the ERK/MAPK and Akt signaling pathways in the CNS (21, 23, 98), and increase the expression of BDNF as well, either the direct activation of these signaling pathways (i.e., direct coupling with activated progesterone receptors) or the regulation of BDNF synthesis and release may be relevant mechanisms that are, at least partially, responsible for progesterone’s protective effects.

## BDNF as a mediator of progesterone’s protective effects

Our laboratory (20, 99) and that of others (100, 101) have shown in various experimental systems, including explants of the cerebral cortex, the injured spinal cord, and in degenerating Wobbler motor neurons, that BDNF expression is increased by progesterone. For instance, a study by Meyer et al., 2013, shows that progesterone promotes upregulation of BDNF in the hippocampus, dentate gyrus, and Cornu Ammonis (CA)3 pyramidal regions of the Wobbler mouse in comparison to vehicle groups (102). Additionally, progesterone treatment has been shown to increase BDNF expression in an animal model of TBI (103). Our lab has shown that in cortical explants, treatment with a physiologically relevant concentration of progesterone (100 nM) for 24 hours induces an approximately 75% increase in both BDNF mRNA and protein expression, an effect that appeared to be consistent with the protective effects of progesterone (20). Furthermore, the principal mediator of the effect of progesterone on BDNF expression was determined to be the “classical” intracellular/nuclear PR, since this effect was inhibited by the pharmacological inhibitor of the PR, RU486, and was lost in PR knockout mice (18). The membrane progesterone receptor (mPR) has also been reported to be potentially involved in promoting expression of BDNF as shown by a study by Castelnovo and Thomas (2022), in which activation of the mPRα in human adipose stem cells differentiated into Schwann cell-like cells (SLC-ASC) led to an upregulation of BDNF expression (104).

The regulation of cell signaling pathways consequent to BDNF action (through interaction with the TrkB receptor) may be an important way by which progesterone elicits its effects, and as such, while synthesis is important, the ability to elicit the release of BDNF is also important. Recently, we have shown that the release of BDNF from glia is triggered by progesterone (19). Interestingly, this release was mediated through a novel membrane-associated progesterone receptor, Pgrmc1, as opposed to the classical PR, which was not involved in this process as our cultured astrocytes lacked the expression of PR (19). Furthermore, this was associated with activation of the ERK5 signaling cascade (19). Based on these findings, our laboratory proposes that the receptor mechanisms of both Pgrmc1 and PR are required to afford sustainable protective effects, since we have shown that the classical PR mediates the effect of progesterone on BDNF expression, and Pgrmc1 appears to mediate the effect of BDNF on release. Specifically, we posit that progesterone, through the PR, replenishes BDNF stores while simultaneously, through Pgrmc1, promotes the release, and thus, availability of BDNF to surrounding cells.

Progesterone-mediated BDNF regulation may also be relevant to progesterone’s cognitive enhancing role. Indeed, numerous studies implicate BDNF in regulating synaptic plasticity in the brain, including LTP (105, 106). Specifically, “late” LTP, a component of LTP that requires *de novo* synthesis of mRNA and protein, appears to require BDNF (107–109). Mechanistically, BDNF is thought to mediate LTP via N-methyl-D aspartic acid (NMDA) receptor phosphorylation, which in turn, alters the function of the receptor. Indeed, BDNF-mediated signaling has been shown to target both the NR1 (110) and the NR2B (111) subunits of the NMDA receptor. Moreover, functional changes in the receptor, such as increasing the open probability of the NMDA receptor channel, are associated with this BDNF-mediated phosphorylation of the NMDA receptor (112, 113). Studies have also shown that signaling pathways elicited by progesterone are implicated in regulating LTP. Specifically, pharmacological or genetic inhibition/disruption of these pathways can inhibit LTP-relevant NMDA receptor phosphorylation (114), BDNF-induced increase in field excitatory postsynaptic potentials (fEPSPs) (115) and produce frank deficits in hippocampal LTP (116). Accordingly, progesterone may regulate LTP (thereby enhancing cognitive function) by direct activation of specific cell signaling pathways consequent to binding to progesterone receptors, or alternatively, influence LTP through the induction of BDNF release, which in turn, activates the signaling pathways that phosphorylate NMDA receptors.

As previously discussed, the ratio of relative abundance of the pro- and mature forms of the neurotrophin may govern the protective effects of synthesized neurotrophins, since pro-neurotrophins preferentially bind to the p75 “pan” neurotrophin receptor to promote cell death, while mature neurotrophins preferentially bind to their cognate Trk receptor and elicit signaling events consistent with cell survival. The laboratory of Dr. Donald Stein has recently reported the expression of pro- versus mature neurotrophins can be differentially regulated by progesterone. Specifically, progesterone treatment led to a decrease in the pro-apoptotic, pro-NGF, while increasing the level of mature NGF in a model of traumatic brain injury (TBI). Although this effect on NGF appears consistent with the protective effects of progesterone, the observed effects of progesterone on pro- versus mature BDNF were not. In fact, progesterone not only decreased the expression of pro-BDNF, but also reduced the expression of mature BDNF and its cognate receptor, TrkB (117). However, recent studies from our lab show that P4 is protective, and that the efficacy of P4’s protective effects was enhanced by inhibiting the microRNA, let-7i (118). Future studies are warranted to help clarify this apparent discrepancy.

## Metabolites of progesterone and their influence on BDNF

When exploring the neuroprotective properties of progesterone, it is crucial to acknowledge the potential involvement of its metabolite, allopregnanolone (3α, 5α tetrahydroprogesterone or THP). This consideration broadens our understanding and opens up new avenues for research into the mechanisms by which progesterone exerts its beneficial effects on brain health. Allopregnanolone, a positive allosteric modulator of the gamma-amino butyric acid-A (GABA-A) receptor, has been shown to have neuroprotective effects by reducing excitotoxicity caused by brain injury or insult (119). There is strong evidence from multiple studies suggesting that allopregnanolone treatment offers substantial benefits in reducing various deficits and consequences associated with traumatic brain injury. Examples of a potential benefit of allopregnanolone is its ability to reduce levels of inflammatory cytokines (120), minimize cell death, and alleviate cognitive deficits (120–125) following traumatic brain injury. It has been postulated that allopregnanolone plays a pivotal role in mediating the protective effects of progesterone. In addition to its other benefits, allopregnanolone has been found to have a significant impact on neurogenesis (refer to the study cited in reference (126) and the accompanying sources). Moreover, extensive research has demonstrated the therapeutic benefits of allopregnanolone in treating Alzheimer’s disease by promoting neurogenesis, enhancing cognitive function and memory, reducing neuroinflammation and beta-amyloid build-up, as well as improving bioenergetics deficits in 3xTgAD mice (127–130). Interestingly, studies have demonstrated that allopregnanolone can exert its protective benefits by modulating BDNF (refer to reference (125) and the relevant citations therein), while the exact method by which allopregnanolone triggers BDNF remains uncertain (i.e., what receptor(s) allopregnanolone interact with). However, noting recent reports that allopregnanolone is a ligand for the PAQR family of membrane progesterone receptors (131), the involvement of multiple classes of membrane progesterone receptors (i.e., to include mPRs and Pgrmc1) in mediating the effect of metabolites of progesterone is indeed emerging.

And while there has been considerable attention to the neuroprotective effects of allopregnanolone, its precursor, 5α-dihydroprogesterone, has also been shown to protect neurons against excitotoxic insults (120, 132, 133). Thus, the protective effects of progesterone may not only be attributed to the abundance of progesterone and its cognate receptors, but also the abundance of the synthetic enzymes responsible for the conversion of progesterone to its neuroprotective metabolites, and in turn, the relatively more recently described receptors associated with their action (e.g., mPR and Pgrmc1).

## Do all clinically used progestins have similar effects on cytoprotection and BDNF?

Medroxyprogesterone Acetate (MPA) is a synthetic progestin often used in conjunction with estrogens to reduce the risk of such cancers that include uterine cancer, associated with unopposed estrogen therapy (134, 135). However, we and others have equivalent effects within the context of their cytoprotective effects. For example, progesterone, but not MPA, was protective against glutamate toxicity in both explants derived from the cerebral cortex (20) and in primary dissociated hippocampal neurons (21). This disparity between the effects of P4 and MPA have also been noted *in vivo*. For example, a study illustrated that the combined administration of estrogen and progesterone in rhesus monkeys protects against coronary vasospasm, whereas the co-administration of MPA with estrogen failed to elicit this protection (136). This difference between progesterone and MPA is also evident in humans, where progesterone administration to post-menopausal women enhanced the protective effects of estrogen on exercise-induced myocardial ischemia, whereas MPA did not (137).

While the discrepancy between the protective effects of progesterone and MPA may be attributed to a variety of factors that include differential regulation of ERK translocation (23), anti-apoptotic protein regulation (21), and calcium homeostasis (21), it appears that a fundamental difference in the regulation of BDNF may also underlie the difference between progesterone and MPA. Notably, our laboratory showed that that while progesterone increased the expression of BDNF in cerebral cortical cultures, MPA suppressed BDNF levels (18).

More recently, the progestin component of a recently FDA-approved contraceptive vaginal ring, segesterone acetate, was shown to have neuroprotective effects (138–140) in the MCAO model of stroke in male rats. Given the preferential affinity of this compound for the PR over other steroid hormone receptors, such data bolster the importance of the classical PR in mediating progesterone’s neuroprotective effects. Further, this study also supports the potential utility of PR-engaging compounds (progesterone and related progestins) in eliciting cytoprotective effects in males as well.

## miRNA and their influence of progesterone’s biological effects

MicroRNAs (miRNAs) are a class of small non-coding RNAs (approximately 20-22 nucleotides in length) that play a major role in regulating gene expression (141). miRNAs were first discovered in *Caenorhabditis elegans*, in which a short RNA sequence produced from the *lin-14* gene downregulated *lin-14* translation (142). miRNAs are initially synthesized as primary-miRNAs (pri-miRNA), a hairpin structure that is predominantly transcribed by the enzyme RNA polymerase II (141). These pri-miRNAs can then undergo RNA editing by adenosine deaminase acting on RNA (ADAR), which can convert adenosine into inosine and potentially modifies cleavage products in succeeding stages of miRNA processing (141, 143). Pre-miRNAs are subsequently produced following cleavage of pri-miRNAs by a microprocessor complex composed of an RNAse III enzyme, Drosha, and the protein DiGeorge syndrome critical region 8 (DGCR8) (141, 144, 145). After these processing steps, pre-miRNAs can then be exported out of the nucleus and into the cytosol through the involvement of exportin 5 (141). Subsequently, pre-miRNAs then undergo an additional modification step in which the RNAse III enzyme, Dicer, binds and cleaves the pre-miRNA to produce a double stranded mature-miRNA duplex (141). Once processed by Dicer, the mature miRNA duplex gets associated with endonucleases belonging to the Argonaute (Ago) family of proteins to form the pre-RNA-induced silencing complex (pre-RISC) (141, 146). Within the pre-RISC, one of the strands of the miRNA duplex, the “passenger strand”, is removed, leaving the single stranded “guide strand” to form the mature RISC complex (146). The RISC complex can bind to the 3’ UTR region of a target mRNA and regulate gene expression through either translation repression or mRNA decay (141, 147). Through these two mechanisms, miRNAs play a major role in regulating numerous genes and various cellular processes. It is estimated that the human genome encodes nearly 2,300 miRNAs, each of which targets about 100 mRNA transcripts (148).

The cellular effects of gonadal steroid hormones, including progesterone, estrogen, and testosterone, have been shown to be influenced by miRNAs. For instance, miRNAs have been shown to negatively regulate the expression of estrogen receptor-α and estrogen receptor-β (149–152), and a study by Epis et al., 2009, suggests that miR-331-3p regulates androgen receptor (AR)-mediated signaling in prostate cancer cell lines (153). As it pertains specifically to progesterone, less is known regarding effects of miRNAs on progesterone in comparison to that of estrogen. However, studies have shown potential miRNA binding sites on progesterone receptor (PR) mRNA that can result in changes in PR expression in breast cancer cell lines and in endometrial carcinogenesis (154–157). Specifically, Gilam et al., 2017, showed that miR-181a, miR-23a, and miR-26b reduced PR expression in ER-positive breast cancer (158). In addition to the classical PR, the influence of miRNAs has been demonstrated on Pgrmc1. Wendler et al., 2010, found that the Pgrmc1 mRNA 3’ untranslated region (UTR) contains a binding site for let7/miR-98, and accordingly, transfection of let-7i in SKOV-3 ovarian cancer cell lines resulted in a decrease in relative Pgrmc1 expression (159). Our laboratory also showed that Let-7i decreases the expression of Pgrmc1 (118). Apart from effects on receptor expression, the release of progesterone has also been shown to be regulated by miRNAs, as evidenced by the study of Sirotkin et al., 2009, where thirty six out of eighty tested miRNA constructs resulted in inhibition of progesterone release from granulosa cells (160, 161). Additionally, transfection of antisense constructs for two of the tested miRNAs resulted in an increase in progesterone release (160, 161). Apart from release, miRNAs have also been shown to regulate progesterone synthesis. Work from An et al., 2020, shows that that transfection of miR-101-3p promotes progesterone synthesis in granulosa cells via inhibition of STC1, which functions to inhibit hCG-stimulated progesterone synthesis, in goat granulosa cells (162). Furthermore, transfection of miR-101-3p results in an increase in mRNA and protein levels of cytochrome P450 family 11 subfamily A member 1 (Cyp11a1) and 3β-hydroxysteroid dehydrogenase (3β-HSD), two enzymes involved in the synthesis of progesterone from cholesterol (162). Collectively, these studies underscore the diagnostic potential of miRNAs in pathological conditions and illustrate miRNAs as a possible therapeutic target for the treatment of various cancers and diseases.

Work from our laboratory has highlighted the therapeutic potential of targeting miRNAs in experimental models of ischemic stroke. We have previously shown that Pgrmc1 is a key mediator in promoting the release of BDNF from cortical astrocytes (19, 163). Based on findings that the miRNA let-7i downregulates expression of Pgrmc1 in peripheral tissue (159), and that the antagomir to let-7f, a sister miRNA to let7i, is neuroprotective in models of ischemic stroke (164), we hypothesized that administration of an antagomir to let-7i, when combined with progesterone, could promote enhanced BDNF release and provide protection in experimental models of ischemic stroke (118). Our findings show that overexpression of let-7i results in downregulation of mRNA and protein levels of Pgrmc1 and BDNF expression in primary cortical astrocytes (118). Furthermore, let-7i was found to inhibit progesterone-mediated BDNF release (118). Additional experiments revealed that treatment of primary neurons with conditioned media from progesterone-treated astrocytes led to an increase in the synaptogenic marker, synaptophysin (SYP) (118). However, SYP expression was greatly diminished in neurons treated with conditioned media from astrocytes treated with let-7i and progesterone, but restored when treated with conditioned media from anti-let7i and progesterone treated astrocytes (118). These results were further validated using an animal model of ischemic stroke, the middle cerebral artery occlusion (MCAo) model, where animals treated with the combination of progesterone and the let-7i antagomir showed a robust increase in mature-BDNF protein levels, significantly reduced infarct sizes, enhanced SYP expression, and improved functional recovery compared to vehicle-treated animals (118). Collectively, these results suggest that inhibition of let-7i in experimental models of stroke promotes neuroprotection by increasing BDNF release, a process mediated by Pgrmc1. These significant findings pave the way for future studies in other disease models in which miRNA dysregulation has been implicated.

## The influence of age on progesterone’s brain-protective efficacy

Aging is related to a decline in circulating gonadal hormone levels, which is more pronounced in women due to menopause. While experimental evidence has shown that estrogen has neuroprotective effects against various insults in animal models, clinical trials such as the Women’s Health Initiative (WHI) have yielded inconsistent results regarding the cognitive benefits of hormone therapy in postmenopausal women. The research conducted in the post-WHI era has yielded compelling evidence that supports the neuroprotective effects of estradiol in animal models of various CNS disorders, including Alzheimer’s disease, Parkinson’s disease, stroke, multiple sclerosis, chronic hypertension, and traumatic brain injury (165–171). Recent findings, however, have raised crucial questions about the circumstances in which hormone therapy after menopause can be beneficial. The effectiveness of hormone therapy may depend on various factors, such as the length of the postmenopausal period before hormone intervention and the subject’s age (172–174). The concept of a therapeutic “window of opportunity” for hormone therapy after menopause has gained traction as a potential explanation for the differing outcomes observed in animal studies and clinical trials. Although the therapeutic potential of estrogens in the brain has been explored to some extent, there is a lack of research on whether a similar limited window of opportunity exists for progesterone and its related metabolite. The remarkable benefits of progesterone in reducing the size of stroke-induced lesions have been observed in young adult (3-month-old) ovariectomized (OVX) C57Bl/6 mice; however, its impact on the neurological outcome seems to be limited when tested on old (12-month-old) OVX mice (175). Such limited information warrants a more comprehensive evaluation of the brain-protective efficacy of progesterone as a function of age and in different models of brain aging and disease.

## Summary

**Figure 1:**
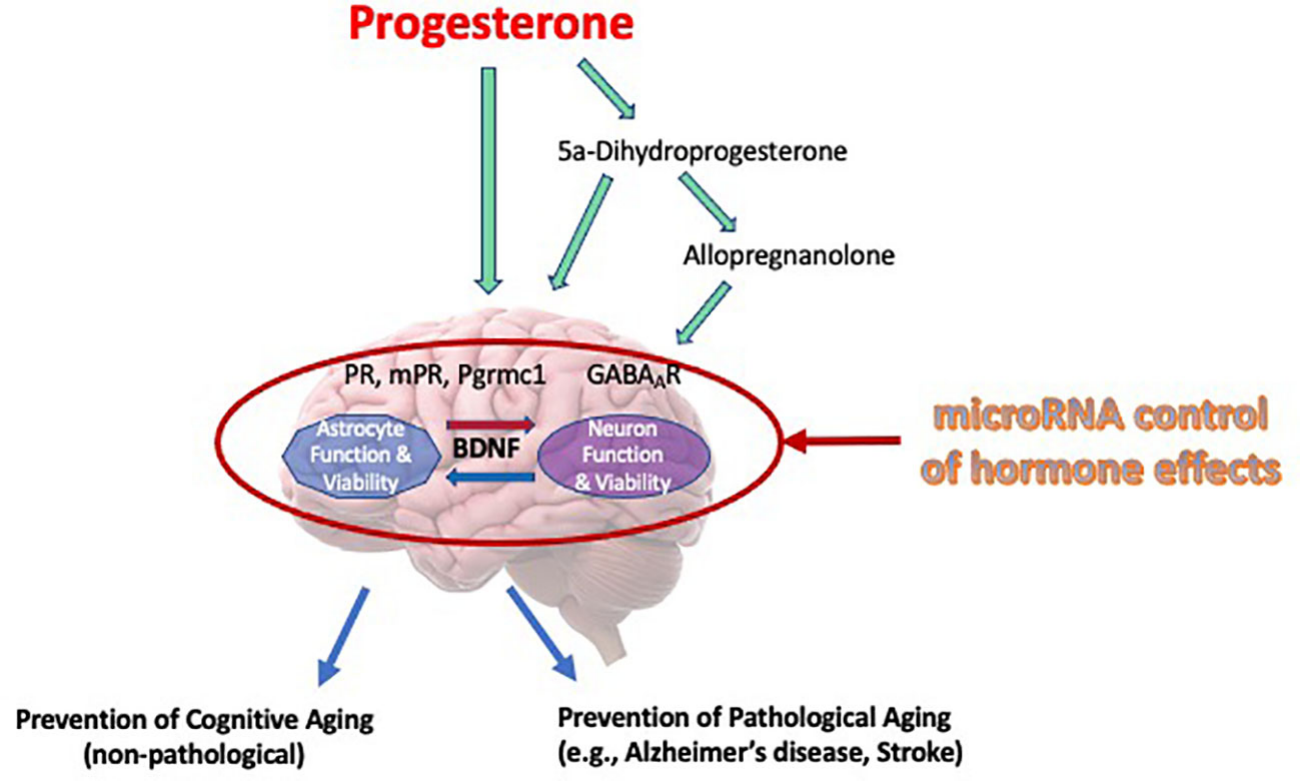
Mechanisms of progesterone’s cytoprotective effects in the brain. Our working model of progesterone’s cytoprotective effects of progesterone is presented in this Figure. It depicts that progesterone, or its metabolites, can elicit its protective influence through various cognate receptors, including allopregnanolone’s effect on regulating neuronal excitability through regulation of GABA-gated current. Moreover, this review article (and thus, this model) depicts brain-derived neurotrophic factor (BDNF) as a central regulator of viability, where it may have bidirectional effects on multiple cell types (i.e., glia and neurons). Further, we recognize that the regulation of progesterone, it’s receptors or mediators of its actions can be regulated by specific miRNA, thus influencing the “cast of characters” that are optimally required to allow progesterone to elicit is neuroprotective effects.

This review has offered information supporting the potential for progesterone as being protective in the brain and recognizing the regulation of BDNF and its associated signaling in this protection. A schematic of our overall working model by which progesterone elicits its neuroprotective efficacy, focusing significantly on BDNF and its associated signaling, and the factors that may alter progesterone’s cytoprotective efficacy, is provided in **Figure 1**. While progesterone regulates the intracellular content of BDNF, the protein and mRNA, as an effect facilitated by the classical progesterone receptor, it also promotes the release of BDNF through the activation of a putative membrane progesterone receptor. Thus, the release of BDNF leads to the activation of TrkB receptors which are positioned on the surface of the adjacent cells, activating a cascade of pro-survival cell signaling pathways which include the ERK/MAPK and PI3/Akt signaling pathways. Remarkably so, the ability of progestin to use neuroprotective properties seems to be associated with its ability to increase BDNF levels. Not only does this association support the important function of BDNF in these protective effects but emphasizes that not all progestins are made identically or equally, especially with respect to their impact on brain function. This awareness, including the key receptor targets of progesterone that relate to various mechanisms that support cell viability, potentially direct future advancement and capability of developing more effective treatments for symptoms of the menopause, and those (neurodegenerative) diseases whose risk or incidence increases during the post-menopausal period.

## Author contributions

MS: Writing – original draft, Writing – review & editing. VK: Writing – original draft, Writing – review & editing. SeK: Writing – original draft, Writing – review & editing. SaK: Writing – original draft, Writing – review & editing. HL: Writing – original draft, Writing – review & editing.
